# Investigation on the Effect of Combined Addition of CNTs and La_2_O_3_ on the Microstructure and Properties of W_2_CoB_2_ Cermet

**DOI:** 10.3390/ma19112378

**Published:** 2026-06-03

**Authors:** Xingyu Zhu, Fan Qu, Yingjun Pan, Deqing Ke, Lian Liu

**Affiliations:** 1 The State Key Laboratory of Refractories and Metallurgy, Wuhan University of Science and Technology, Wuhan 430081, China; 2 State Key Laboratory of Special Materials Surface Engineering, China Academy of Machinery Wuhan Research Institute of Materials Protection Co., Ltd., Wuhan 430030, China

**Keywords:** vacuum liquid phase sintering, W_2_CoB_2_ cermet, microhardness, fracture toughness, transverse rupture strength, wear

## Abstract

W_2_CoB_2_ is a ternary boride-based cermet. Featuring high hardness, high melting point, excellent wear resistance and corrosion resistance, it has been widely used in numerous industrial fields such as cutting processing, surface protection and mold manufacturing. Toughening is a major issue that needs to be addressed for ceramic materials. In this study, the toughness of cermets is improved by the combined addition of CNTs and La_2_O_3_. The W_2_CoB_2_ cermets were fabricated via vacuum sintering, and the effects of CNTs and La_2_O_3_ on the microstructure and properties of the cermets were systematically investigated. The microstructure and phase composition of the specimens were characterized using a SEM and X-ray diffractometry (XRD), respectively. The density of the specimens was measured by the Archimedes drainage method. A Vickers microhardness tester was employed to determine the microhardness and fracture toughness of the specimens. The transverse rupture strength was tested using an electronic universal testing machine, while the wear resistance was evaluated via a wear tester. The results indicate that the addition of either CNTs or La_2_O_3_ can refine the grain size and improve the toughness of the cermets. The simultaneous incorporation of CNTs and La_2_O_3_ further enhances grain refinement and mitigates the issue of uneven dispersion of CNTs in the specimens. When 0.5 wt.% CNTs and 0.3 wt.% La_2_O_3_ are added, the specimen exhibits the following optimal properties: a density of 9.33 g/cm^3^, a microhardness of 2046 HV_0.5_, a fracture toughness of 12.36 MPa·m^1/2^, a transverse rupture strength of 985 MPa, and a friction coefficient reduced to 0.36. Synergistic addition of CNTs and La_2_O_3_ achieves grain refinement and uniform microstructure, which significantly improves the friction and wear performance and service stability of the cermet. The material retains high hardness and wear resistance, accompanied by enhanced comprehensive mechanical and service properties. Further studies will aim to cut costs while preserving material performances, facilitating its industrial application.

## 1. Introduction

Although WC-Co cemented carbides are widely used in metal processing and cutting applications owing to their high hardness, excellent wear resistance, and favorable fracture toughness, WC undergoes oxidation when the temperature exceeds 800 °C [1,2]. This oxidation leads to a significant reduction in both hardness and corrosion resistance of the material. Consequently, WC-Co cemented carbides still fail to meet the service requirements under relatively extreme conditions. Therefore, the development of novel cemented carbide materials has become an inevitable trend in current research and industrial advancement [3].

Ternary boride cermets exhibit high hardness and superior wear resistance comparable to those of WC-Co cemented carbides, while also possessing excellent high-temperature stability. Thus, such cermet materials serve as promising alternatives to WC-Co cemented carbides. Among ternary boride cermets, W_2_CoB_2_ demonstrates extremely high hardness and good wear resistance, enabling it to operate in environments with temperatures up to 1000 °C [4,5]. However, W_2_CoB_2_ suffers from relatively high brittleness, making the toughening of W_2_CoB_2_ a key research focus in the field [6,7,8].

Carbon nanotubes (CNTs) are recognized as the most attractive and ideal reinforcements for fabricating high-performance composite materials, attributed to their exceptional properties including ultra-high elastic modulus, high strength, and low density. The high aspect ratio of CNTs allows them to exert a toughening effect in cermets through crack propagation regulation; meanwhile, they can refine grains, thereby enhancing the hardness and strength of the material [9,10,11]. Zhang et al. prepared Ti(C, N) cermets with a multi-walled carbon nanotube (MWCNT) addition of 0.4 wt.% via spark plasma sintering (SPS) [12]. Their findings revealed that a small amount of carbon atoms in MWCNTs would diffuse into the lattice of Ti(C, N), which improved the interfacial bonding performance between MWCNTs and the cermet matrix. This interfacial enhancement ultimately contributed to the simultaneous improvement of the cermet’s strength and toughness. Additionally, Wang et al. achieved in-situ synthesis of CNTs within the powder via chemical vapor deposition (CVD) and subsequently prepared dense WC-CoCr coatings using high-velocity air fuel (HVAF) spraying technology [13]. Their study indicated that strong metallurgical bonding was formed between CNTs and both the WC hard phase and the CoCr binder phase; the addition of 0.3 wt.% CNTs was found to simultaneously enhance the hardness and fracture toughness of the WC-CoCr coating.

Rare earth oxides are characterized by ultra-high melting points and excellent high-temperature stability; more importantly, they can inhibit the abnormal grain growth of cermet grains, thereby significantly optimizing the overall performance of cermets [14,15,16,17]. Zhou et al. investigated the effect of different La_2_O_3_ addition contents on the microstructure of Ti(C, N) cermets [18]. The results showed that La atoms would diffuse into the ceramic phase, leading to a reduction in grain size. Elgazzar et al. prepared Ti(C, N) cermets through vacuum sintering and systematically studied the influence of La_2_O_3_ on the cermets’ microstructure and mechanical properties [19]. Their results demonstrated that an appropriate amount of La_2_O_3_ could effectively refine the cermet grains and significantly improve the microstructure homogeneity and mechanical properties of the cermet. In contrast, an excessive addition of La_2_O_3_ would result in a significant decrease in the transverse rupture strength (TRS) of the cermet.

Both CNTs and rare earth oxides have been proven to significantly improve the microstructure and mechanical properties of cermets [20,21,22,23]. However, there remains a paucity of studies focusing on the combined addition of CNTs and rare earth oxides for cermet toughening. Therefore, in this study, CNTs and La_2_O_3_ were simultaneously incorporated into cermets to investigate their combined effects on the microstructure and properties of the cermets, with a specific focus on exploring the synergistic strengthening and toughening mechanisms. This research is expected to provide a new approach and experimental basis for the toughening of cermets, and holds potential application value for the industrialization of W_2_CoB_2_-based cermets.

## 2. Experiment

### 2.1. Preparation

**Figure 1:**
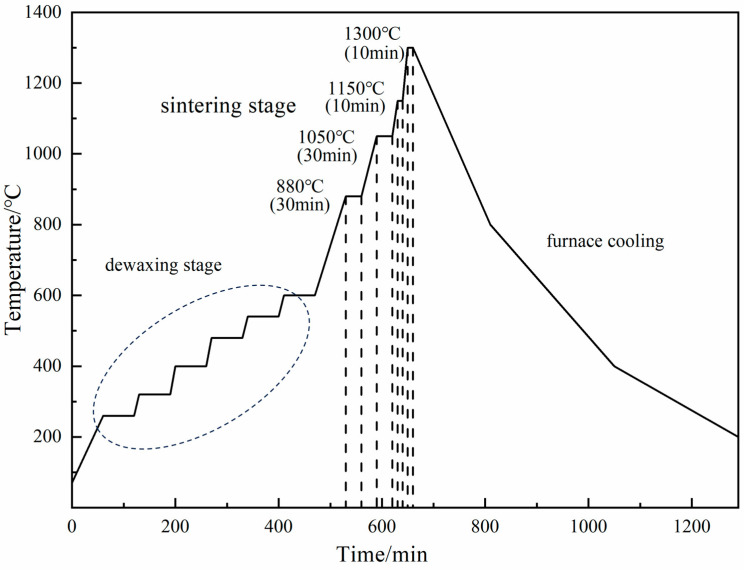
Heating curves of cermets [24].

The powders were prepared by mixing in a QM-1SP4 ball mill for 72 h, followed by suction filtration and drying (Nanjing Chishun Technology Development Co., Ltd., Nanjing, China). To ensure the structural integrity of CNTs, CNTs are added in the final 1 h of ball milling. The proportions of the powders are shown in Table 1. CNTs and La_2_O_3_ were extra additives to the initial powder ratio. After passing through an 80-mesh sieve, the powders were pressed into shape and then sintered using a ZT-15-20 vacuum sintering furnace at 1300 °C (Shanghai Chenhua Electric Furnace Co., Ltd., Shanghai, China). The vacuum degree during sintering was maintained below 1 Pa, and the specific sintering process is shown in Figure 1.

### 2.2. Test

Phase analysis of the samples was conducted using an X’Pert Pro MPD system (PANalytical B.V., Almelo, The Netherlands). The microstructure and morphology of the samples were observed with a field-emission scanning electron microscope (FESEM, Nova 400 Nano SEM, FEI, Hillsboro, OR, USA). The hardness of the samples was measured using an HX-500 Vickers hardness tester (Laizhou YuTong Test Instrument Co., Ltd., Laizhou, China). The density of the samples was determined via the Archimedes drainage method. The fracture toughness of the samples was measured with an HXS-1000A Vickers hardness tester (Laizhou YuTong Test Instrument Co., Ltd., Laizhou, China). Grain size measurements were performed on SEM images via Image-Pro 6. Tribological properties were tested at room temperature of 25 °C using a UMT-2 high-load scratch tester (Bruker, Billerica, MA, USA). A YG8 cemented carbide ball with a diameter of 10 mm and Rockwell hardness of 90 HRC was adopted as the friction pair. The applied load was 20 N, sliding distance 4 mm, sliding speed 15 mm/s, and test duration 120 min.

The Evans equation is a classic semi-empirical formula for the indentation fracture toughness of brittle materials. Specifically, it is used to calculate the Mode I fracture toughness of materials through Vickers indentation test data, and is applicable to typical brittle materials such as ceramics and glasses [25].

$$K_{IC}\phi /HV\sqrt{a}=0.15k{\left(c/a\right)}^{-3/2}$$

In the simplified formula, *ϕ* is the constraint factor (≈3), and when c/a is relatively large, the value of k is 3.2. The following formula is obtained after simplification.

$$K_{IC}=0.16HVa^{2}c^{-3/2}$$

## 3. Results

### 3.1. XRD

**Figure 2:**
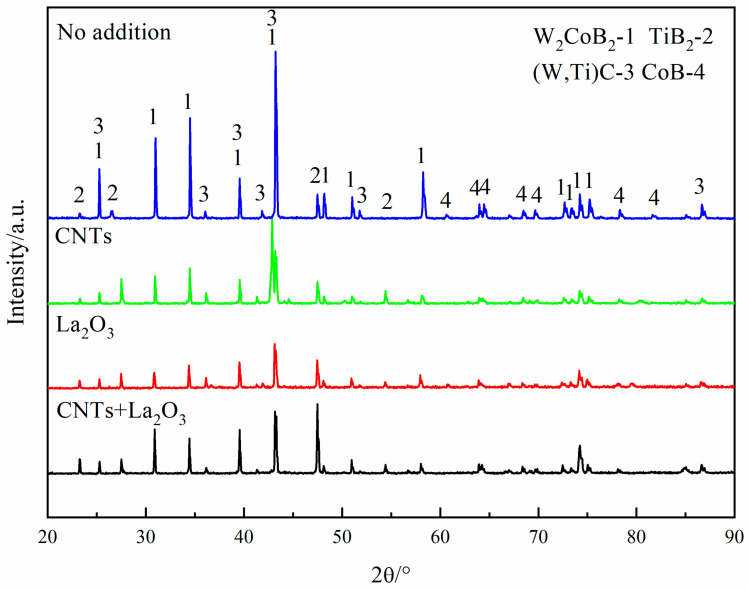
X-ray diffraction patterns of the cermet samples.

Figure 2 shows the XRD patterns of the samples before and after the addition of CNTs and La_2_O_3_. During the sintering process, the sample undergoes the reaction 2WC + 2TiB_2_ + 3Co = W_2_CoB_2_ + 2TiC + 2CoB. It can be observed that the addition of either CNTs or La_2_O_3_ does not affect the phase composition of the sample. However, after the addition of CNTs, the relative peak intensities of W_2_CoB_2_ and (W,Ti)C increase, which indicates that the addition of CNTs can promote the progress of the in-situ reaction.

**Figure 3:**
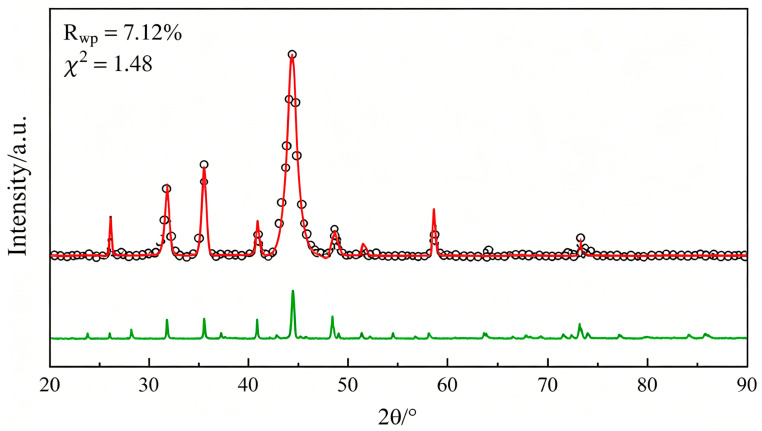
XRD patterns analyzed via Rietveld refinement.

Figure 3 shows the Rietveld-refined XRD patterns. The crystallographic structures of orthorhombic W_2_CoB_2_ (Cmcm, No. 63), CoB (Pnma, No. 62), cubic (W,Ti)C (Fm3m, No. 225), and metallic Co (Fm3m, No. 225) were employed as the initial models.

Refinement was carried out stepwise: background, scale factor, zero shift, lattice parameters, peak shape, crystallite size, and microstrain were optimized sequentially until convergence. The final phase mass fractions were 80.52 wt.% W_2_CoB_2_, 9.68 wt.% CoB, 7.35 wt.% (W,Ti)C, and 2.45 wt.% residual Co, with R_wp_ = 7.12% and χ^2^ = 1.48, indicating a reliable fit.

### 3.2. Microstructure

**Figure 4:**
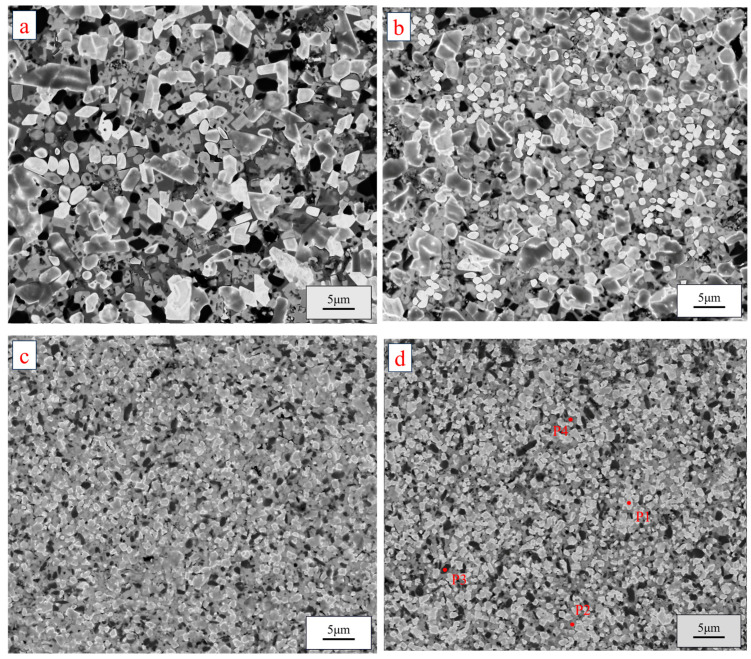
Microstructure of (**a**) S1, (**b**) S2, (**c**) S3 and (**d**) S4 [24].

**Figure 5:**
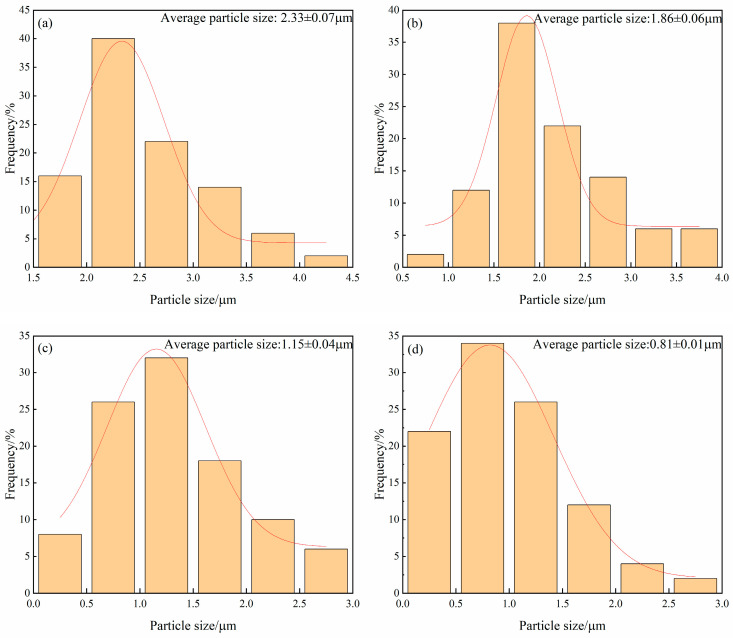
Histogram of grain size frequency distribution of (**a**) S1, (**b**) S2, (**c**) S3 and (**d**) S4.

Figure 4 and Figure 5 show the microstructural images and grain size distribution diagrams of the samples before and after the addition of CNTs and La_2_O_3_. Table 2 presents the EDS point scan elemental composition and atomic percentage of different microstructures in Figure 4d. The average grain sizes of S1, S2, S3, and S4 are 2.33 μm, 1.86 μm, 1.15 μm and 0.81 μm, respectively. It can be seen from the figures that after the addition of CNTs or La_2_O_3_, the average grain size decreases significantly by 20.2% and 50.6%, respectively, with La_2_O_3_ exhibiting a more remarkable grain refinement effect. After the combined addition of CNTs and La_2_O_3_, the average grain size further decreases by 65.2%, and the grain refinement effect is more uniform with a more concentrated grain size distribution.

After the addition of CNTs, nucleation is more likely to occur near the CNTs, forming a large number of fine grains. Moreover, a network structure is formed around the grains to inhibit grain growth, thereby achieving grain refinement. After doping, La_2_O_3_ remains at the grain boundaries and hinders the migration of grain boundaries, thus suppressing grain growth and realizing grain refinement. Before the addition of CNTs and La_2_O_3_, there are a large number of irregularly shaped grains in the ceramic, which is a manifestation of the anisotropy of grain growth. This phenomenon may be caused by the uneven temperature gradient during the heating process. After the addition of CNTs and La_2_O_3_, the number of elongated grains decreases. This is because CNTs and La_2_O_3_ are uniformly distributed in the sample, so grains are more likely to nucleate and grow around CNTs and La_2_O_3_, increasing the number of grains and grain boundaries and making the grains equiaxed.

The composition of each microstructure can be determined from the atomic percentages in Table 2 combined with XRD results. The W:Co ratio in the P1 microstructure is approximately 2:1, so it may be W_2_CoB_2_. The W:Ti ratio in the P2 microstructure is close to 3:1, so it may be a (W,Ti)C solid solution. The P3 microstructure contains a large amount of Ti, so it may be TiB_2_ and TiC. The P4 microstructure contains a large amount of Co and a small amount of Ti, so it may be the Co binder phase dissolved with a portion of TiC.

### 3.3. Properties

#### 3.3.1. Density and Microhardness

**Figure 6:**
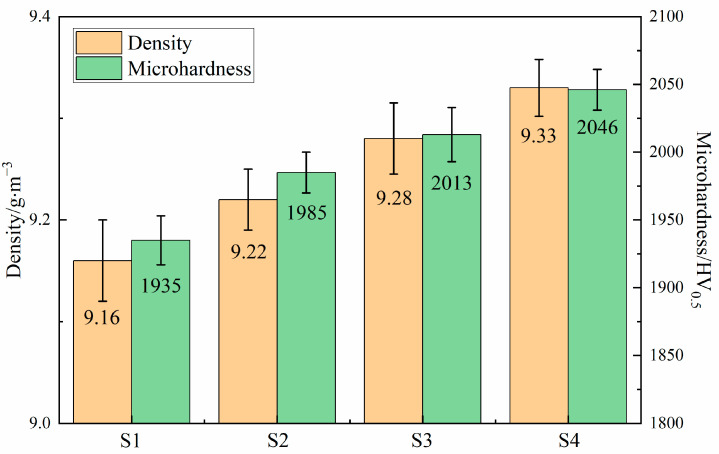
Density and microhardness of the different samples.

Figure 6 shows the comparison diagram of density and hardness of the samples before and after the addition of CNTs and La_2_O_3_. The theoretical density of the sintered cermet was calculated to be 9.44 g/cm^3^ based on the mass fractions of each constituent phase obtained from Rietveld refinement. The actual density of the samples is determined using the formula ρ = m/(V_2_ − V_1_), where m is the mass of the sample in a dry state, and V_2_ − V_1_ is the volume difference of the liquid medium before and after the sample is immersed. Combined with Archimedes-measured bulk density, the total porosity of the cermet was quantitatively determined to be approximately 1.17%.

During the sintering process, pores will form, leading to a decrease in actual density. However, after the addition of CNTs alone, La_2_O_3_ alone, and their combination, the density of the samples increases by 0.66%, 1.31%, and 1.86% respectively, while the microhardness increases by 2.58%, 4.03%, and 5.74% respectively. Ke et al. prepared WCoB cermets with an optimal density of 9.27 g/cm^3^ via vacuum sintering [26]. In this study, the optimal density of the as-prepared cermets reached 9.33 g/cm^3^, representing an increase of 0.6%, which is close to the theoretical density of 9.37 g/cm^3^. Zhang et al. prepared NbC/WCoB cermet cladding layers by laser cladding, with an optimal Vickers hardness of 1862.5 HV_0.5_ [27]. The optimal Vickers hardness of the cermets prepared in this study is 2046 HV_0.5_, representing an increase of 9.85% in comparison.

CNTs are uniformly distributed in the samples, and their tubular structure can better discharge the gas generated during the sintering process, thereby increasing the sample density. During sintering, the high surface energy of CNTs promotes the massive formation of fine grains near the CNTs, significantly reducing the grain size. Meanwhile, CNTs pin at the grain boundaries and hinder plastic deformation, which improves the sample hardness.

After the addition of La_2_O_3_, the sintering temperature can be reduced, allowing the hard phase to distribute more uniformly in the Co binder phase and reducing the number of pores caused by sintering shrinkage. At the same time, La_2_O_3_ can refine grains and increase the number of grain boundaries, thereby dispersing pores and improving the sample density. La_2_O_3_ is dispersedly distributed at the grain boundaries, exerting a dispersion strengthening effect. Additionally, it pins the grain boundaries and inhibits abnormal grain growth, achieving a grain refinement strengthening effect, thus further improving the sample hardness. The combined addition of CNTs and La_2_O_3_ superimposes their respective strengthening effects; meanwhile, La_2_O_3_ promotes the more uniform distribution of CNTs in the samples, resulting in a more significant grain refinement strengthening effect.

#### 3.3.2. Fracture Toughness

**Figure 7:**
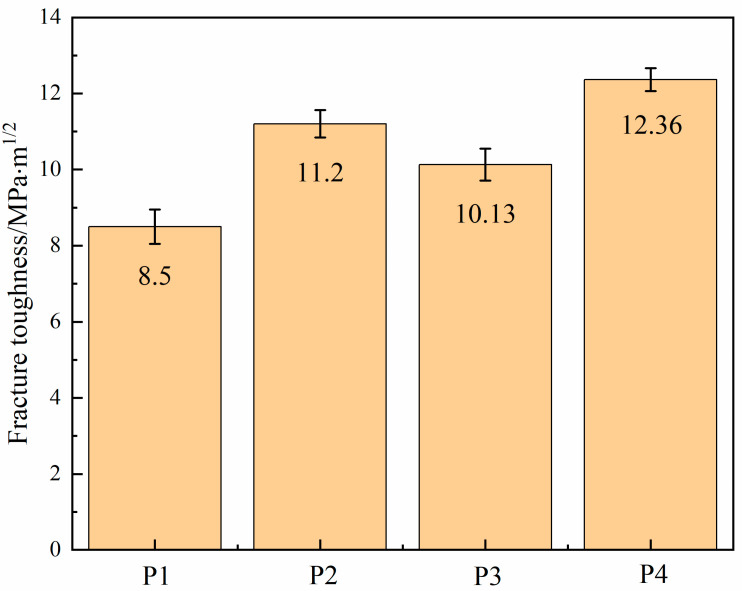
Fracture toughness of the different samples.

Figure 7 shows the comparison diagram of fracture toughness of the samples before and after the addition of CNTs and La_2_O_3_. The addition of CNTs alone, La_2_O_3_ alone, and their combination all significantly improve the fracture toughness of the samples, with increases of 31.8%, 19.2%, and 45.4% respectively. Zhang et al. prepared NbC/WCoB cermet coatings via laser cladding, with an optimal fracture toughness of 8.65 MPa·m^1/2^ [27].The optimal fracture toughness of the cermets fabricated in this study is 12.36 MPa·m^1/2^, representing an increase of 42.9% compared with the reported result.

CNTs are uniformly distributed in the samples. When a crack propagates and comes into contact with CNTs, the CNTs exert a crack pinning effect on the crack, preventing its direct propagation. When the crack can no longer propagate forward, it deflects at the location of the CNTs; this deflection increases the energy required for crack propagation, thereby enhancing the fracture toughness of the sample.

La_2_O_3_ segregates at the grain boundaries, forming a grain boundary pinning effect. By inhibiting grain growth, it significantly refines the grain size of the material and increases the number of grain boundaries. The increased grain boundaries, in turn, increase the frequency of crack deflections, thereby hindering crack propagation and improving the fracture toughness of the sample.

The combined addition of CNTs and La_2_O_3_ can inhibit the structural damage of CNTs in high-temperature environments. Under the effect of synergistic toughening and strengthening, the toughening effect becomes more stable, which further explains the highest fracture toughness increment observed in the composite-added sample.

#### 3.3.3. Transverse Rupture Strength

**Figure 8:**
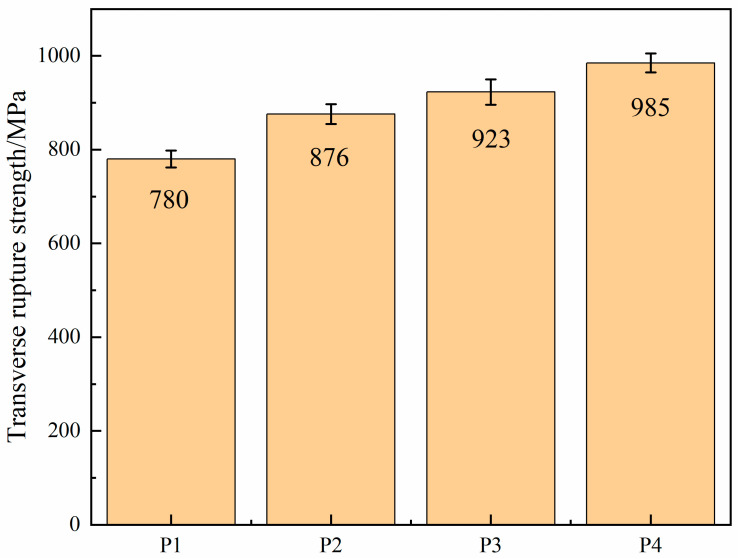
Transverse rupture strength of the different samples.

Figure 8 shows the comparison diagram of transverse rupture strength of the samples before and after the addition of CNTs and La_2_O_3_. The addition of CNTs alone, La_2_O_3_ alone, and their combination all improve the transverse rupture strength of the samples, with increases of 12.3%, 18.3% and 26.3% respectively.

After the addition of CNTs, due to their extremely high aspect ratio and high mechanical strength, they can bear greater stress when the sample is subjected to external forces, reducing stress concentration in the sample and thus increasing the transverse rupture strength of the sample.

In cermets, La_2_O_3_ can improve the wettability between the hard phase and the Co binder phase, enhance their interfacial bonding performance, and exert a significant grain refinement strengthening effect on ceramic grains. According to the Hall–Petch equation (σ = σ_0_ + kd^−1^/^2^), where σ is the material strength, σ_0_ is the lattice friction stress, k is the Hall–Petch coefficient, and d is the average grain size, the refined grain size can significantly increase the transverse rupture strength of the sample.

When CNTs and La_2_O_3_ are added in combination, La_2_O_3_ can promote the more uniform distribution of CNTs in the sample, reduce CNT agglomeration, and at the same time enhance the interfacial bonding between CNTs and the ceramic matrix phase, thereby further improving the strengthening efficiency of CNTs and increasing the transverse rupture strength.

#### 3.3.4. Fracture Morphology

**Figure 9:**
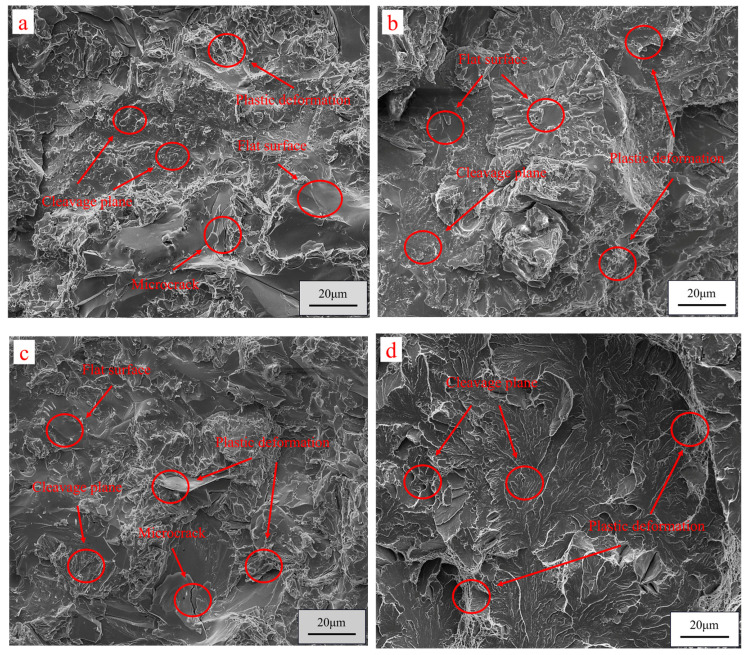
Fracture morphologies of (**a**) S1, (**b**) S2, (**c**) S3 and (**d**) S4.

Figure 9 shows the fracture morphology images of the samples before and after the addition of CNTs and La_2_O_3_. All samples exhibit a typical brittle fracture morphology, which is attributed to the extremely high brittleness of the W_2_CoB_2_ hard phase. In the fracture morphology of S1, relatively large and flat cleavage planes can be observed, with a small number of microcracks around them. The residual stress generated by the shrinkage of the green compact during the sintering process cannot be fully eliminated, leading to the formation of these microcracks under external stress during fracture.

The Co binder phase undergoes plastic deformation during the fracture process. With the addition of CNTs alone, La_2_O_3_ alone, and their combination, the plastic deformation degree of the binder phase in the fracture morphology gradually increases. From the fracture morphology of S4, a large number of river patterns formed by cleavage steps can be seen, with tear ridges formed by the plastic deformation of the Co binder phase around them.

Cermets in this study consist of two key phases: the hard phase and the binder phase. As confirmed by XRD and EDS analysis, W_2_CoB_2_ is the primary hard phase with high hardness and high brittleness, while Co serves as the metal binder phase to improve toughness. During the fracture process, the W_2_CoB_2_ hard phase fractures along cleavage planes to form a smooth fracture morphology, and the Co binder phase undergoes plastic deformation to form tear ridges.

CNTs form good interfacial bonding with the Co binder phase and distribute uniformly in the sample after addition. When the sample fractures, CNTs exhibit a pull-out strengthening and toughening effect. La_2_O_3_ exhibits an excellent grain refinement effect and increases the number of grain boundaries; its addition significantly enhances the roughness of the fracture surface. The simultaneous addition of CNTs and La_2_O_3_ exerts a synergistic toughening effect, which significantly improves the toughness of the sample and increases the number of tear ridges formed by the plastic deformation of the Co binder phase.

#### 3.3.5. Wear

**Figure 10:**
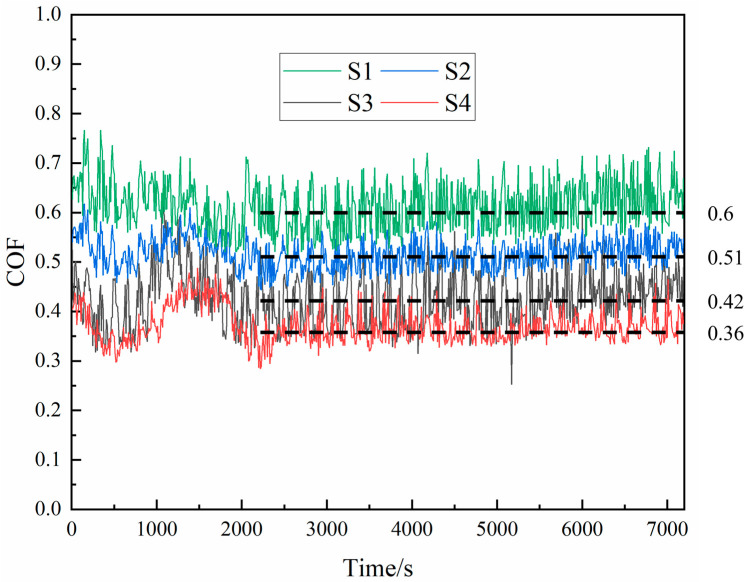
COF of the different samples.

Figure 10 shows the variation curves of the friction coefficient (COF) of the samples before and after the addition of CNTs and La_2_O_3_. The friction coefficient curves of different samples (S1–S4) can be divided into three distinct stages: initial wear stage, running-in stage, and steady wear stage [28,29].

The first stage is the initial wear stage. When the load is applied to the wear surface, the Co binder phase deforms plastically, and a small amount of the hard phase fractures and detaches. Under the action of the load, the detached hard phase particles form three-body abrasion on the wear surface, resulting in an abnormal increase in the friction coefficient. With the addition of CNTs alone, La_2_O_3_ alone, and their combination, the interfacial bonding performance between the hard phase and the Co binder phase is enhanced, the amount of detached hard phase is reduced, and the effect of three-body abrasion is significantly weakened.

The second stage is the running-in stage. As three-body abrasion proceeds, the Co binder phase on the wear surface is gradually peeled off, and the friction coefficient decreases gradually. Since W_2_CoB_2_ is a hard phase with high hardness and high brittleness, the friction coefficient fluctuates significantly during the running-in period. Among the samples, S1 shows the most obvious COF fluctuation due to the poor bonding between the hard phase and binder phase.

The third stage is the steady wear stage. As wear continues, three-body abrasion decreases gradually, and the Co binder phase deforms plastically under the action of the load, making the variation of the friction coefficient tend to be stable gradually. The addition of CNTs and La_2_O_3_ improves the toughness of the Co binder phase; thus, in the steady wear stage, the friction coefficients of S2, S3 and S4 are stabilized in a smaller range. Meanwhile, the simultaneous addition of CNTs and La_2_O_3_ makes the microstructure of the sample more uniform, resulting in the smallest fluctuations in the friction coefficient of S4 during the steady wear stage.

**Figure 11:**
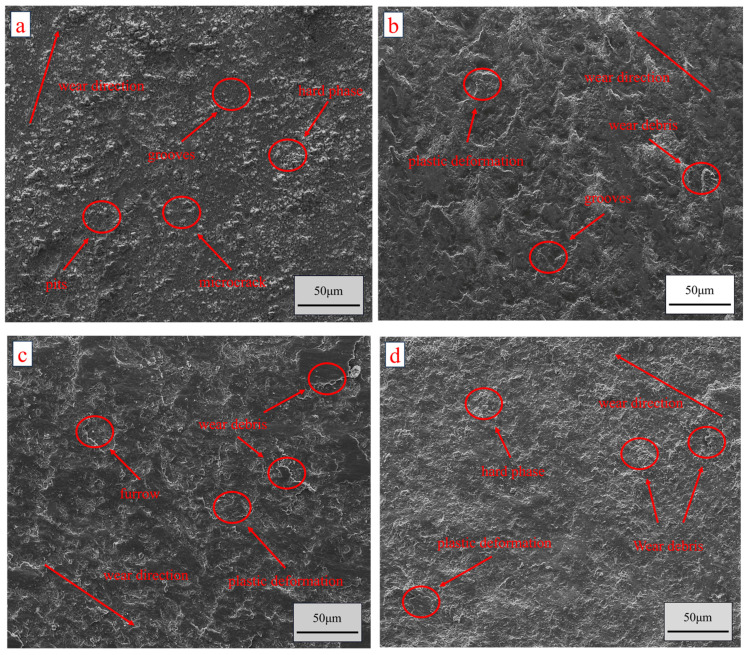
Wear morphologies of (**a**) S1, (**b**) S2, (**c**) S3 and (**d**) S4.

The wear morphologies are shown in Figure 11. Obvious cracks, pits, and granular debris can be observed in S1. When a load is applied, the Co binder phase in the sample deforms plastically under stress, while the hard phase plays a supporting role. As the load increases, a small amount of the hard phase fractures. With the progression of wear, the detachment of the hard phase particles creates pits; under the action of the load, abrasive wear forms on the surface, and the Co binder phase is cut to form grooves during the wear process. As wear continues, a large amount of the surface Co binder phase is consumed, the grooves become shallower, and cracks appear simultaneously.

In S2, plastic deformation features along the wear direction can be seen. During wear, CNTs are uniformly distributed in the Co binder phase and play a supporting role, preventing massive peeling of the Co binder phase. This, in turn, protects the hard phase and reduces its detachment. At the same time, the plasticity of the Co binder phase increases; after peeling, it adheres to the wear surface and forms adhesive wear under the load.

A large number of pits can be observed in S3. The addition of La_2_O_3_ refines the grains of the hard phase and enhances the interfacial bonding between the hard phase and the Co binder phase. During wear, a small amount of the Co binder phase peels off and adheres to the wear surface, forming a new wear surface that deforms along the wear direction. As wear proceeds, this new surface gradually cracks under shear stress to form pits.

In S4, the wear morphology is observed to be relatively flat, with fewer pits formed by the detachment of the hard phase. The synergistic strengthening effect of CNTs and La_2_O_3_ can significantly improve the mechanical strength of the hard phase and the toughness of the Co binder phase. During wear, the detachment of the hard phase particles and the peeling of the Co binder phase are significantly reduced.

## 4. Toughening Mechanism

**Figure 12:**
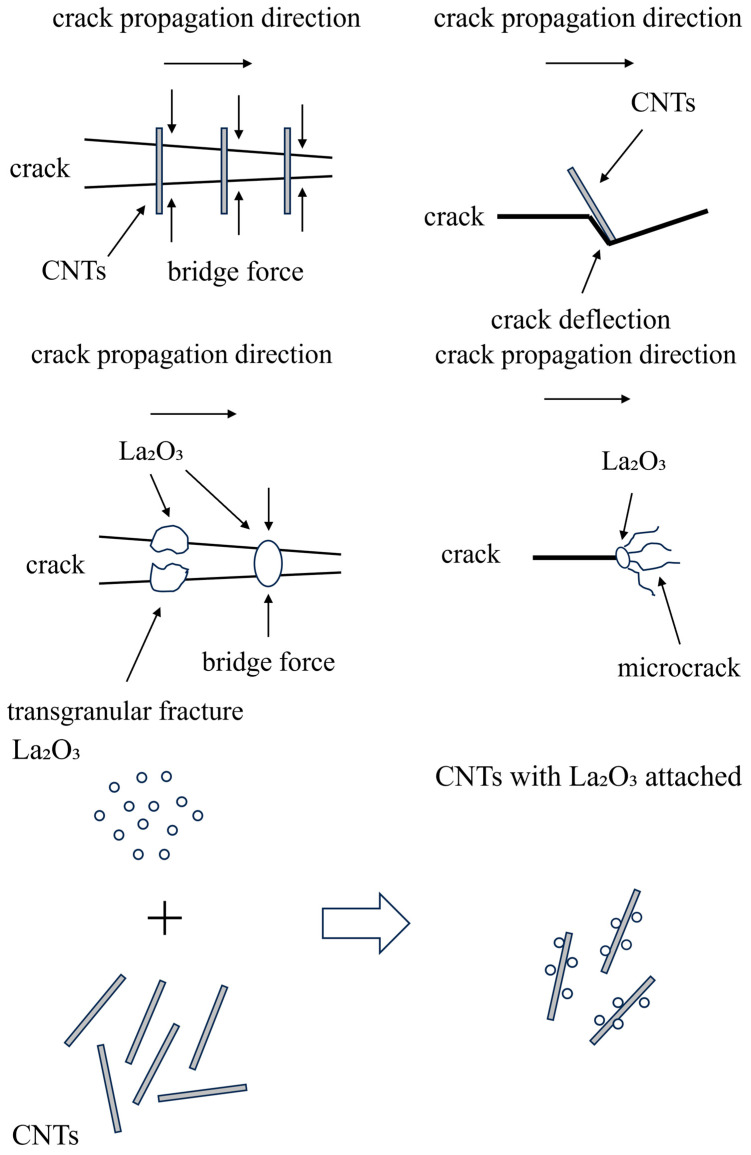
Toughening mechanism of the sintered sample.

The schematic diagram of the toughening mechanism of CNTs and La_2_O_3_ on cermets is shown in Figure 12. Both CNTs and La_2_O_3_ exhibit a grain refinement effect on cermets, increasing the number of grain boundaries and thereby improving the mechanical strength of cermets. After dispersion treatment, CNTs are uniformly distributed in the Co binder phase; when cracks propagate and encounter CNTs, crack bridging and deflection occur. Specifically, CNTs bear the tensile stress generated during crack propagation, slowing down the crack growth rate and forming stable crack bridging. When the tensile stress exceeds the interfacial bonding force between CNTs and the Co binder phase, CNTs are gradually pulled out—this process consumes a large amount of fracture energy and thus effectively hinders crack propagation. Due to the high aspect ratio and elastic modulus of CNTs, cracks propagating through CNTs may extend along the axial direction of CNTs, deviating from their original propagation path and significantly reducing the efficiency of crack growth.

La_2_O_3_ is uniformly distributed in cermets and can act as a second-phase particle to strengthen and toughen the material. When cracks encounter dispersedly distributed La_2_O_3_ particles, La_2_O_3_ bears the tensile stress of crack propagation and slows down the crack growth rate. When the tensile stress is excessively high, La_2_O_3_ particles fracture, resulting in transgranular fracture that disperses local stress and reduces crack propagation speed. When La_2_O_3_ is highly dispersed, it induces multi-directional stress on the main crack, leading to the formation of a large number of microcracks around the main crack. These microcracks disperse the energy required for main crack propagation, thereby further hindering crack growth.

When CNTs and La_2_O_3_ are added simultaneously, La_2_O_3_ adsorbs on the surface of CNTs, which improves the dispersibility of CNTs in the Co binder phase and reduces the occurrence of CNT agglomeration [30]. This synergistic effect allows CNTs and La_2_O_3_ to exert a better combined toughening effect, which explains the highest fracture toughness increment of S4 in Figure 7.

## 5. Conclusions

By studying the effects of adding CNTs and La_2_O_3_ on the microstructure and properties of W_2_CoB_2_, the following conclusions are drawn:

1. Both CNTs and La_2_O_3_ can effectively reduce the grain size, and their simultaneous addition can further enhance the grain refinement effect.
2. The addition of CNTs and La_2_O_3_ can significantly improve the density and microhardness of the cermet. The density is increased by 0.66% and 1.31% respectively, and can reach 9.33 g/cm^3^ when added in combination; the microhardness is increased by 2.58% and 4.03% respectively, and can reach 2046 HV_0.5_ when added in combination.
3. The addition of CNTs and La_2_O_3_ improves the fracture toughness, transverse rupture strength, and wear resistance of W_2_CoB_2_ cermet. The fracture toughness is increased by 31.8% and 19.2% respectively, and reaches the optimal value of 12.36 MPa·m^1/2^ when added in combination; the transverse rupture strength is increased by 12.3% and 18.3% respectively, and reaches the optimal value of 985 MPa when added in combination. The synergistic strengthening effect of CNTs and La_2_O_3_ reduces the friction coefficient from 0.6 to 0.36.

## 6. The Outlook

This work proves that CNTs and La_2_O_3_ enhance the toughness of W_2_CoB_2_ cermets without sacrificing hardness. Future investigations will concentrate on precise composition, advanced technology, mechanism exploration and application expansion. Supported by interdisciplinary research, key problems such as hardness-toughness balance, defect regulation and mass production will be solved. This material will progressively substitute WC-Co cemented carbides and play a vital role in high-end equipment and extreme-condition protection.

## Figures and Tables

**Table 1:** Powder composition ratio of the samples.

	WC/wt.%	TiB_2_/wt.%	Co/wt.%	CNTs/wt.%	La_2_O_3_/wt.%
S1	59	21	20	-	-
S2	59	21	20	0.5	-
S3	59	21	20	-	0.3
S4	59	21	20	0.5	0.3

**Table 2:** Atomic percentage of elements at each point in Figure 4d.

s	W	Co	Ti
P1	61.19	34.23	4.58
P2	65.75	8.89	25.36
P3	3.58	9.52	86.9
P4	5.52	80.88	13.6

## Data Availability

The original contributions presented in this study are included in the article. Further inquiries can be directed to the corresponding authors.
